# New Properties of Supramolecular Complexes and Drug Nanoparticles

**DOI:** 10.3390/pharmaceutics18010136

**Published:** 2026-01-22

**Authors:** Elena V. Uspenskaya, Anton V. Syroeshkin

**Affiliations:** Department of Pharmaceutical and Toxicological Chemistry, Medical Institute, Peoples’ Friendship University of Russia Named After Patrice Lumumba (RUDN University), 6 Miklukho-Maklaya St., Moscow 117198, Russia; syroeshkin-av@rudn.ru

## 1. Introduction

Supramolecular chemistry—is “the chemistry beyond the molecule bearing on the organized entities of higher complexity that result from the association of two or more chemical species held together by intermolecular forces”—J.-M. Lehn (Nobel Prize lecture), 1987.

**Figure 1:**
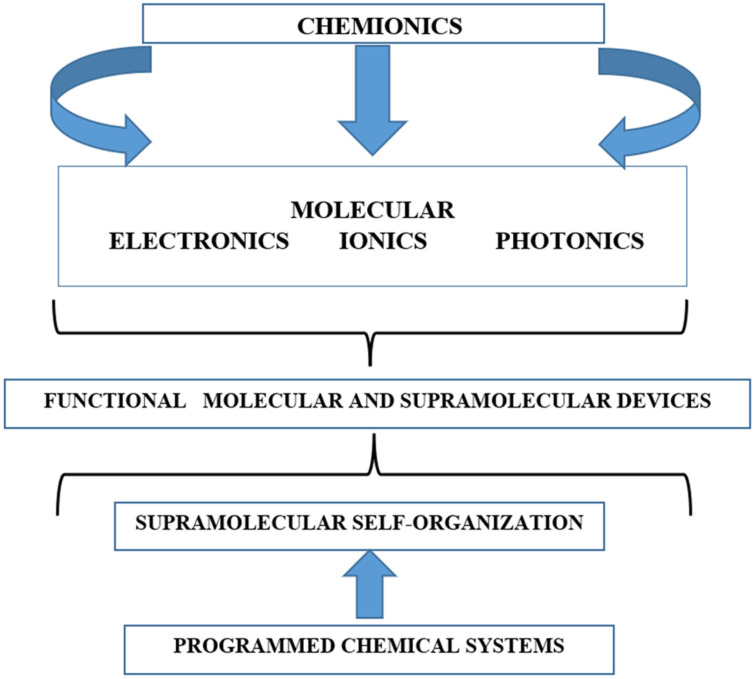
The concept of chemionics, governed by photo-, electro- and ion-active molecular components “from below” and by the programmable properties of self-organized supramolecular systems “from above” [3].

The term “Chemistry beyond the molecule” as a designation for supramolecular chemistry was introduced by Jean-Marie Lehn more than half a century ago and was intended to unify those branches of physical chemistry that describe the properties of ensembles of “supermolecules” [1,2]. In the view of J.-M. Lehn, who shared the Nobel Prize jointly with C.J. Pedersen and D.J. Cramp, a distinctive feature of supermolecules as organized systems with non-covalent interactions is their ability to create molecular devices for the transmission and transformation of signals and information involving photons, electrons, and ions, giving rise to the concept of chemionics (Figure 1).

The presented scheme (see Figure 1) illustrates the direction of development of molecular and supramolecular devices for information processing and signal generation. The functions of supramolecules include molecular recognition, catalysis, transport, and the handling of information carried by the interacting structures—the ligand and the molecular receptor [4].

The Special Issue “New Properties of Supramolecular Complexes and Drug Nanoparticles” is devoted to the current state of supramolecular chemistry, and specifically to highly ordered entities—biologically active supramolecular complexes and self-assembled nanoscale ensembles held together by cooperative and synergistic non-covalent interactions (NCIs) (hydrophobic interactions, π–π stacking, multiple hydrogen bonding, van der Waals surfaces) [5]. Although the energy of such interactions per mole is relatively low (0.1–5.0 kcal·mol^−1^), their collective behavior plays a vital role in fundamental biological processes [6]. The studies presented, in line with the aims and scope of the SI, are intended to open up new opportunities in the application of supramolecular systems as pharmaceutical agents [7].

Over the lifetime of this SI, 17 scientific papers have been published, including 12 original research articles and 5 reviews. The authors of the submitted manuscripts are leading experts in the field of self-organized supramolecular systems and are affiliated with various international laboratories and research centers.

## 2. An Overview of the Published Articles

The collected manuscripts discuss the synthesis, preparation, characterization of properties, and practical applications of supramolecular systems and nanomaterials of various origins.

The aim of the study reported in [Contribution 1] was to employ magnetic nanoparticles for the detection of circulating tumor cells (CTCs); the authors demonstrate approaches to the synthesis and surface functionalization of magnetic nanoparticles that enable efficient initiation of ligand–receptor interactions, leading to the formation of supramolecular complexes with CTCs.

In [Contribution 2], lipophosphonoxins (LPPOs), a novel group of broad-spectrum antibacterial compounds, are reviewed with a focus on their structure–activity relationships, mechanisms of action, toxicity, and resistance. The molecular design and pharmaceutical formulations of amphiphilic LPPO, which form pores and supramolecular aggregates within biological membranes, are described. Such coatings effectively suppress *S. aureus* infections on wound surfaces and accelerate healing without impairing fibroblast or keratinocyte function, while exhibiting low systemic exposure in plasma and liver at therapeutic concentrations, which is characteristic of topically applied agents. 

Contribution 3 describes the development of nanocatalysts (polymeric, inorganic, and photosensitive) to enhance the generation of highly reactive OH·radicals and suppress tumor cell growth. Fenton reaction-based therapy is demonstrated as a promising cancer treatment strategy. Further optimization of nanoparticle synthesis, reaction control, and safety assessments will advance this field toward fully harnessing its potential against malignant tumors. 

Contribution 4 provides a pharmaceutically oriented overview of their broad biological activities. The authors emphasize multicentric complexes that integrate non-covalent and coordination interactions.

Contribution 5 analyzes homogeneous core–shell hybrid nanoparticles and anisotropic nanoparticles (Janus, multicompartment, patchy) operating via the substrate-drug interaction. Anisotropic (Janus, multicompartment) supramolecular particles offer functional compartmentalization, enabling selective binding of drugs with distinct physicochemical properties—for instance, doxorubicin (DOX) forming a hydrophobic core while the hydrophilic component localizes in another domain.

In Contribution 6, the relationship between chitosan-based polymer blends (with polyethylene glycol, polyvinyl alcohol, and polyvinylpyrrolidone) and their antimicrobial activity against *Staphylococcus aureus* is investigated. Chitosan systems with functionalizing agents form supramolecular complexes via non-covalent interaction networks, altering macroscopic properties (structure, swelling, release) and antibacterial activity (bacterial adhesion).

Contribution 7 demonstrates how nanoparticles generate intrinsic radiothermal emission—a highly specific phenomenon suitable for the quality control of pharmaceutical nanomedicines containing biologically active entities (such as virus-like particles, antibodies, interferons, and humic/fulvic acids).

Contribution 8 focuses on the structural and supramolecular aspects of riluzole salts with dihydroxybenzoic acids. Structural parameters, particularly the positioning of OH groups, govern the self-assembly of supramolecular architectures, motifs, and lattice energetics.

pH-responsive Fe_3_O_4_@CaCO_3_ nanocomposites with high doxorubicin loading for cancer treatment, reported in Contribution 9, enable accelerated drug release and enhanced cytotoxicity in tumor microenvironments. This hierarchical core–shell system features a supramolecular porous CaCO_3_ matrix retaining DOX via electrostatic, hydrogen-bonding, and coordination interactions, with magnetic Fe_3_O_4_ cores for external field control. 

Contribution 10 develops a biocompatible, water-soluble macrocyclic platform based on an amino-substituted pillararene for specific binding to extracellular DNA, inhibiting pathogenic *Staphylococcus aureus* biofilms. It highlights the role of long-range interactions and supramolecular phase architecture in modulating biofilm matrix mechanics and diffusion.

Contribution 11 examines selenium nanoparticles (SeNPs) as nanodispersed supramolecular entities whose physicochemical interactions with IgG immunoglobulins elicit immune responses distinct from those induced by ionic or molecular selenium forms. Dose-dependent effects on granulocyte viability defined a therapeutic window for immunomodulation, with spectral changes confirming SeNP-IgG complex formation and local protein conformational rearrangements. 

The biological activity of selenium supramolecular compounds is further explored in Contribution 12, which reports a novel selenium–taxifolin (Se–TAX) nanocomplex exhibiting neuroprotective efficacy under ischemia/reoxygenation. Taxifolin functionalizes SeNPs, forming a core–shell supramolecular structure confirmed by optical and spectroscopic methods, yielding synergistic cytoprotection beyond individual components.

Contribution 13 addresses supramolecular drug systems of humic acids complexed with poorly soluble antivirals. Humic acid systems show distinct second-order scattering quenching in the presence/absence of guest molecules (e.g., mangiferin, favipiravir), indicating stable complex formation.

Contribution 14 investigates solid dispersions of microcrystalline lactose saturated with ultra-high dilutions of active pharmaceutical ingredients, which form supramolecular states with specific THz emission. THz spectroscopy enables non-destructive quality control of these organized drug carriers.

Contribution 15 describes the development of protocols for isolating humic substance-based polyelectrolytes and for characterizing their properties and virucidal activity. Hierarchical supramolecular organization correlates with structure–activity relationships, exemplifying emergent antiviral properties in self-assembling nanodispersions distinct from parent materials.

In Contribution 16, the authors systematically studied cosolvency, micellization, and cyclodextrin complexation affecting the solubility/permeability of a novel antifungal compound. Supramolecular organization distinguishes solvation from structural solvent effects; micelle incorporation sharply increases solubility, while cyclodextrin yields characteristic solubility-concentration profiles, stabilizing the promising agent.

The final contribution (Contribution 17) describes highly diluted anti-interferon-gamma (IFNγ) antibody solutions that modify structural properties of IFNγ aqueous solutions remotely via water-fluidized lactose, which can be detected by terahertz time-domain spectroscopy (THz-TDS). External vibrational exposure with sequential dilutions induces metastable supramolecular organization through novel solvent–substance interactions.

## 3. Conclusions/Future Directions

Advances in the study of new properties of medicinal agents that exist as supramolecular systems and/or nanoparticles confirm their importance and potential for modern pharmacy [8]. The unique properties of supramolecular chemistry objects, which exhibit intermolecular long-range effects and spatiotemporal control over their self-assembly, provide fundamentally new opportunities for the targeted design of drug products with tunable selectivity, bioavailability, and pharmacological profiles. Taken together, these factors create a conceptual basis for the development of personalized medicine and pharmacy [9,10,11]. The results under discussion outline the future of pharmaceutics, in which precision, efficacy, and patient-centeredness will be at the forefront of drug development grounded in the unique properties of such “supermolecules.”

## Abbreviations

SI	Special Issue
NCIs	Non-covalent interactions
CTCs	Circulating Tumor Cells
LPPO	Lipophosphonoxins
DOX	Doxorubicin
PEG, PVA, PVP	Polyethylene Glycol, Polyvinyl Alcohol, Polyvinylpyrrolidone
VLP	Virus-like particles
SeNPs	Selenium nanoparticles
TAX	Taxifolin
HAs	Humic acids
HS	Humic Substances
THz	Terahertz Frequency Range
THz-TDS	Terahertz time-domain spectroscopy
GDs	Gradualized drugs
IFNγ	Interferon-gamma
